# Complementary ab initio and X-ray nanodiffraction studies of Ta_2_O_5_

**DOI:** 10.1016/j.actamat.2014.10.006

**Published:** 2015-01-15

**Authors:** R. Hollerweger, D. Holec, J. Paulitsch, M. Bartosik, R. Daniel, R. Rachbauer, P. Polcik, J. Keckes, C. Krywka, H. Euchner, P.H. Mayrhofer

**Affiliations:** a Christian Doppler Laboratory for Application Oriented Coating Development at the Institute of Materials Science and Technology, Vienna University of Technology, A-1040 Vienna, Austria; b Department of Physical Metallurgy and Materials Testing, Montanuniversität Leoben, A-8700 Leoben, Austria; c Institute of Materials Science and Technology, Vienna University of Technology, A-1040 Vienna, Austria; d Oerlikon Balzers Coating AG, LI-9496 Balzers, Liechtenstein; e Plansee Composite Materials GmbH, D-86983 Lechbruck am See, Germany; f Department Materials Physics, Montanuniversität Leoben and Materials Center Leoben, A-8700 Leoben, Austria; g Ruprecht Haensel Laboratory, University of Kiel, Leibnizstrasse 19, D-24098 Kiel and Helmholtz-Zentrum Geesthacht, Institute for Materials Research, D-21502 Geesthacht, Germany

**Keywords:** Ta_2_O_5_, Structure, Nanobeam diffraction, Ab initio, DOS

## Abstract

The complex structure of Ta_2_O_5_ led to the development of various structural models. Among them, superstructures represent the most stable configurations. However, their formation requires kinetic activity and long-range ordering processes, which are hardly present during physical vapor deposition. Based on nano-beam X-ray diffraction and concomitant ab initio studies, a new metastable orthorhombic basic structure is introduced for Ta_2_O_5_ with lattice parameters *a *= 6.425 Å, *b *= 3.769 Å and *c *= 7.706 Å. The unit cell containing only 14 atoms, i.e. two formula unit blocks in the *c* direction, is characterized by periodically alternating the occupied oxygen site between two possible positions in succeeding 002-planes. This structure can be described by the space group 53 (*Pncm*) with four Wyckoff positions, and exhibits an energy of formation of −3.209 eV atom^−1^. Among all the reported basic structures, its energy of formation is closest to those of superstructures. Furthermore, this model exhibits a 2.5 eV band gap, which is closer to experimental data than the band gap of any other basic-structure model. The sputtered Ta_2_O_5_ films develop only a superstructure if annealed at temperatures >800 °C in air or vacuum. Based on these results and the conveniently small unit cell size, it is proposed that the basic-structure model described here is an ideal candidate for both structure and electronic state descriptions of orthorhombic Ta_2_O_5_ materials.

## 1 Introduction

Tantalum pentoxide (Ta_2_O_5_) is an important semiconductor material exhibiting a high dielectric constant and refractive index, and therefore it is commonly used for capacitors or optical coatings [1], [2]. Furthermore, Ta_2_O_5_ exhibits a wealth of other beneficial properties such as outstanding good biocompatibility [3], corrosion resistance [2], [4] and piezoelectric behavior [5], [6].

The complex crystal structure of Ta_2_O_5_, which determines its properties, has been carefully studied for more than 50 years [7], [8]. In one of the first studies, Lehovec [7] attributed an orthorhombic structure to tantalum pentoxide, which is nowadays known as β-Ta_2_O_5_. The additionally observed peaks within his X-ray diffraction (XRD) patterns originate from superstructures like those described by Grey et al. [9], Audier et al. [10] or Stephenson and Roth [11]. Lagergren [12] showed a reversible phase transformation of the orthorhombic β-Ta_2_O_5_ to a tetragonal high-temperature phase (α-Ta_2_O_5_) at ∼1320 °C. In addition, a hexagonal metastable low-temperature phase (δ-Ta_2_O_5_) has been reported [13], which itself irreversibly transforms into β-Ta_2_O_5_ at elevated temperatures. A highly characteristic property of low-temperature Ta_2_O_5_ structures is the position of oxygen ions, which is exactly between two tantalum ions in the *c* direction, but varies greatly in the *a* and *b* directions.

Nevertheless, most reported Ta_2_O_5_ crystal structures are closely related to each other. For example, a simple monoclinic cell with a structural angle *γ* = 120° equals a hexagonal cell, which itself contains a base-centered orthorhombic lattice with an *a*/*b* ratio of $1/\sqrt{3}$. The orthorhombic structure can easily transform into a tetragonal structure as the *b* and *c* lattice constants of both structures are similar (3.66 vs. 3.88 Å). This already indicates that small variations in the ion positions can easily influence the resulting structure of Ta_2_O_5_ and hence its properties. Consequently, this could be the reason for a large variety of different reported basic and super-structures. For more details on the various crystal structures, the reader is referred to the review article by Hummel et al. [14].

Based on cross-sectional X-ray nano-diffraction studies of reactively sputtered Ta_2_O_5_ thin films in combination with density functional theory (DFT) calculations, a new orthorhombic Ta_2_O_5_ basic-structure model is proposed. Its unit cell, containing only 14 atoms, allows an easier description of the orthorhombic low-temperature Ta_2_O_5_ phases compared with the superstructures, and thus it contributes to the understanding of the formation of various low-temperature structures as well as their transformation to the superstructures.

## 2 Experimental

Tantalum oxide films were deposited at 500 °C on (1 0 0) oriented silicon substrates (20 × 7 × 0.35 mm^3^) on a laboratory scale, magnetically unbalanced, Leybold Heraeus magnetron sputtering system. The metallic tantalum target (99.95% purity, 75 mm diameter) was DC sputtered using an Ar/O_2_ glow discharge at a total pressure of 0.4 Pa and an Ar/O_2_ flow ratio of 0.5. Further details on the deposition conditions and the experimental setup can be found in Ref. [15].

Isothermal vacuum annealing treatments for 1 h at temperatures up to 1000 °C were performed at a base pressure of ⩽5 × 10^−4 ^Pa using a heating and cooling rate of 20 and 50 K min^−1^, respectively. Corresponding ambient air annealing treatments were conducted in a Nabertherm furnace.

The chemical composition of the films was obtained by elastic recoil detection analysis (ERDA) using 35 MeV Cl^7+^ incident ions and the analyzing procedure after Barrada et al. [16].

Nanoindentation was carried out with a UMIS indentation system (Fischer Cripps Laboratories) using a Berkovich indenter tip. Hardness and indentation modulus were obtained by evaluating load–displacement curves after Oliver and Pharr [17]. To guarantee minimized substrate interference, the normal loads ranged only between 2 and 10 mN, which yielded a maximum penetration depth <10% of the film thickness.

Structural investigations were performed by XRD using a Bruker D8 equipped with a Cu *K*_α_ radiation source (*λ* = 1.54 Å) and Sol-X detector in Bragg Brentano geometry. Detailed XRD studies of the films across the layer thickness were obtained by X-ray nano-diffraction using the nano-focus end-station of P03 micro- and nanofocus X-ray scattering (MINAXS) beamline at Petra III synchrotron (Hamburg, Germany). The diameter of the X-ray beam was <500 nm, and the wavelength λ was 0.808 Å. Further experimental details on this advanced technique are reported in Refs. [18], [19], [20], [21], [22]. The growth morphology of the coatings was investigated by surface and cross-section scanning electron microscopy (SEM) studies with Zeiss EVO50 and FEI XL30 instruments operated at an acceleration voltage of 15 kV. To avoid surface charging, a thin Au layer was deposited onto the samples prior to these investigations.

Several Ta_2_O_5_ structures were modeled by the Vienna ab initio simulation package [23], [24] using projector augmented wave pseudopotentials [25] and the generalized gradient approximation (GGA) [26]. The plane wave cut-off energy of 800 eV and more than 2000 *k*-points·atom ensure a total energy accuracy of 1 meV atom^−1^. Volume, cell-shape, and all ion positions were allowed to fully relax during the calculations. For the Lehovec model, the cell shape needed to be fixed to keep the orthorhombic structure during relaxation.

The “visualization for electronic and structural analysis” software [27] was used to visualize the various crystal structures.

## 3 Results and discussion

### 3.1 Film deposition and characterization

**Fig. 1:**
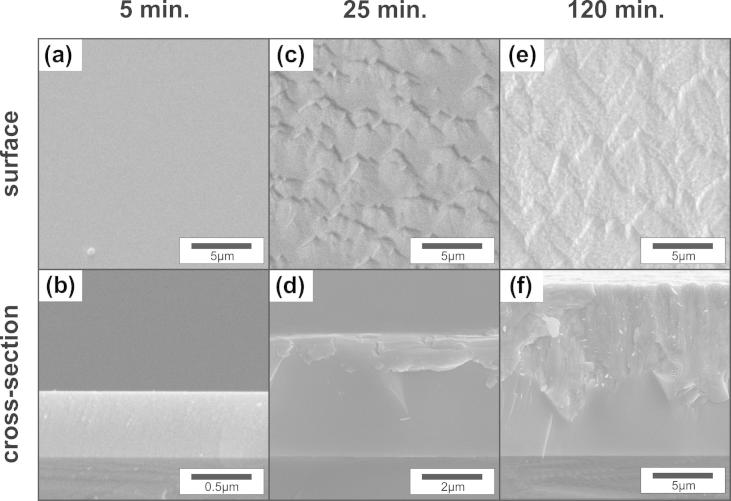
Surface and cross-sectional SEM micrographs of Ta_2_O_5_ thin films after deposition times of (a, b) 5 min, (c, d) 25 min and (e, f) 120 min, respectively.

**Fig. 2:**
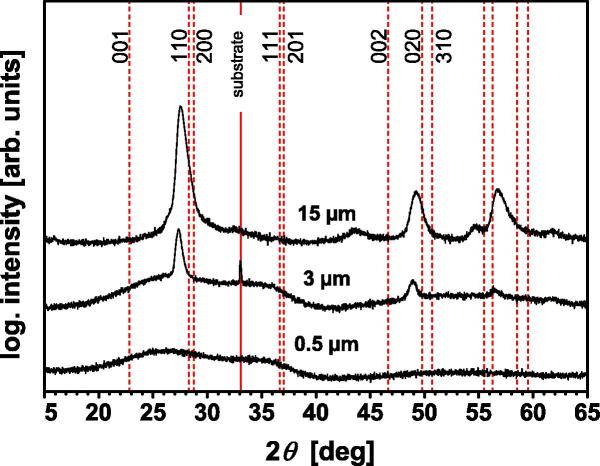
XRD patterns of ∼0.5, 3 and 15 μm thin Ta_2_O_5_ films. The dashed red vertical lines indicate the peak positions of the orthorhombic low-temperature β-Ta_2_O_5_ structure [28] after Lehovec [7]. (For interpretation of the references to colour in this figure legend, the reader is referred to the web version of this article.)

After a very short deposition time of only 5 min, the Ta_2_O_5_ film exhibits a smooth surface (Fig. 1(a)) and a dense featureless morphology (Fig. 1(b)). If the deposition time is increased to 25 min, the formation of small islands can be identified in the surface SEM image (Fig. 1(c)) and the corresponding fracture cross section (Fig. 1(d)). Further increasing the deposition time to 120 min leads to a pronounced increase in the surface roughness (Fig. 1(e)). Also the corresponding fracture cross-section in Fig. 1(f) clearly reveals two distinct growth morphologies. The substrate near region is almost featureless, whereas a fibrous-like structure develops with progressing film growth. The XRD patterns of these three Ta_2_O_5_ films with different thicknesses of ∼0.5 μm, ∼3 μm and ∼15 μm clearly show that the crystalline character evolves with increasing film thickness (Fig. 2). According to the structural description by Lehovec [7] and the derived powder diffraction file [28], the films develop a highly (1 1 0)–(2 0 0) oriented orthorhombic Ta_2_O_5_ structure. The pronounced offset of the XRD peak positions—more than 0.5° below that of the powder diffraction file [28]—suggests a highly strained lattice in the *a* and *b* directions. However, this XRD pattern could also fit the hexagonal structures published by Khitrova and Klechkovskaya [29], [30] or Fukumoto and Miwa [31]. (The hexagonal structures can also be described by an orthorhombic structure, as presented later in Table 1 on discussing the ab initio calculations.)

**Table 1:** Lattice parameters, energy of formation *E*_f_ and band-gap^a^ of various Ta_2_O_5_ structures; the individual lattice parameters are normalized to two formula units in the *c* direction; all presented values were calculated by DFT in this work for a comprehensible comparison.

Structure	*a* (Å)	*b* (Å)	*c* (Å)	Volume (Å^3^ atom^−1^)	*E*_f_ (eV atom^−1^)	Band gap (eV)^a^
Orthorhombic β′-Ta_2_O_5_	6.425	3.769	7.706	13.339	−3.209	2.5
Orthorhombic oxygen deficient β′-Ta_2_O_5_	6.462	3.722	7.654	13.811	−3.146	2.5
Orthorhombic after Stephenson and Roth [11]	6.270	3.759	7.648	12.878	−3.259	1.5
Orthorhombic after Lehovec [7]^b^	6.297	3.724	7.894	13.224	−2.947	0
Hexagonal after Fukumoto and Miwa [31]^c^	7.341	–	7.780	12.965	−3.006	1
Monoclinic^d^	6.837	3.071	7.988	11.651	−2.962	1

a The band gap is obtained from density of states calculations, presented in Fig. 9.

b To keep the orthorhombic structure, the cell shape needed to be fixed during relaxation.

c This hexagonal cell can also be described as an orthorhombic cell with lattice parameters of a = 6.357 Å, *b *= 3.670 Å and *c* = 7.780 Å (for two formula units in the *c* direction).

d If the orthorhombic subtraction-type-model based on U_3_O_8_[33] is relaxed (allowing also for cell shape changes), a monoclinic structure is obtained with the inclined axis-angle of 104°.

**Fig. 3:**
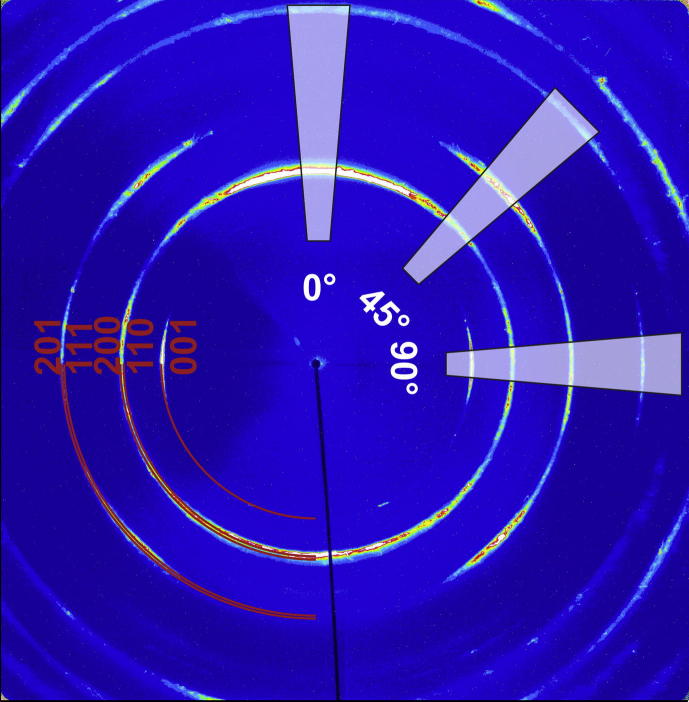
Summed-up nano-diffraction pattern of all 2-D diffraction patterns recorded along the cross-section of ∼15 μm thin Ta_2_O_5_ film. The highlighted segments at azimuth angles *δ* = 0°, 45° and 90° indicate the areas used to obtain the nano-diffraction patterns as a function of the film thickness, which are presented in Fig. 4.

More detailed XRD studies as a function of the film thickness (in 100 nm steps) were carried out by cross-sectional X-ray nano-diffraction. Fig. 3 shows a representative two-dimensional (2-D) diffraction pattern constructed by summing up all 2-D patterns recorded across the coating thickness. The Debye–Scherrer rings clearly indicate an orthorhombic Ta_2_O_5_ structure (in good agreement with Fig. 2). There are no characteristic signs for a hexagonal structure or an orthorhombic superstructure. The strong variation in the intensity along the diffraction rings suggests a pronounced (1 1 0)–(2 0 0) fiber texture. The highlighted cake segment of the Debye–Scherrer rings at an azimuth angle *δ* of ∼0°, with only (1 1 0) and (2 0 0) diffraction rings, corresponds to the diffraction on crystallographic planes oriented approximately parallel to the substrate surface. This segment, which corresponds to the conventional XRD patterns collected in Bragg–Brentano configuration, is in excellent agreement with Fig. 2. The highlighted segment at *δ* = 90°—representing diffraction on planes oriented perpendicular to the film–substrate interface—reveals the presence of all major diffraction planes of the orthorhombic Ta_2_O_5_ structure. Consequently, the XRD results (conventional and X-ray nano-diffraction) show that the Ta_2_O_5_ films grew with the *c*-plane perpendicular to the substrate surface (the 001 and 002 orientations can only be detected for *δ* = 90°).

**Fig. 4:**
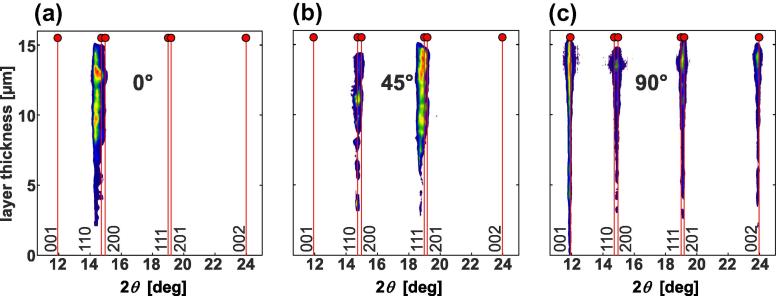
Evolution of the nano-diffraction patterns with film thickness. These patterns are obtained from the three cake segments at azimuth angles of (a) *δ* = 0°, (b) 45° and (c) 90°, highlighted in Fig. 3. The vertical lines indicate the peak positions for the orthorhombic “Lehovec” structure [28].

Fig. 4(a), (b) and (c) shows the development of the nano-diffraction patterns as a function of the film thickness for *δ* = 0°, 45° and 90°, respectively. The used segments, indicated in Fig. 3, cover an azimuthal angle width of Δ*δ* = 10°. Within the first ∼0.5 μm of the film growth, no crystalline diffraction can be detected for *δ* = 0° (see Fig. 4(a)). This is in agreement with conventional XRD of the ∼0.5 μm thin film (Fig. 2), and with transmission electron microscopy (TEM) investigations (not shown here), which also indicate an amorphous structure. In this ∼0.5 μm growth region, only a 001-orientation of an orthorhombic structure can be detected for an azimuthal angle of *δ* = 90° (see Fig. 4(c)). For the following growth region from ∼0.5 to 3 μm, 110 and 200 orientations are also detectable for *δ* = 0°. Consequently, these results also agree with conventional XRD of the ∼3 μm thin Ta_2_O_5_ films (see Fig. 2). With a further increase in the film thickness from ∼3 to ∼15 μm (i.e. the maximum thickness), the crystalline nature becomes more pronounced. The results clearly document that, after ∼0.5 μm, the Ta_2_O_5_ film exhibits an orthorhombic structure throughout the film thickness, with pronounced 110 and 200 growth orientations. Overall, the patterns agree with the orthorhombic structure proposed by Lehovec [7]. This counts especially for lattice planes oriented perpendicular to the substrate surface represented by the azimuth angle *δ* = 90° (Fig. 4(c)). However, the lattice planes oriented parallel to the substrate surface (Fig. 4(a) and (b)) exhibit a rather large offset. As this offset does not change across the film thickness (see Fig. 4), it is envisioned that the films crystallize in a modified Lehovec structure rather than in a strained structure. In the case of first-order strains, one would expect a changing peak offset with increasing film thickness owing to varying strains. Further explanations for the formation of a modified Lehovec structure are provided later in Section 3.2, where the ab initio calculations are discussed. The Lehovec model is derived from the major diffraction peaks of the more accurate description of a stable Ta_2_O_5_ superstructure. Owing to the necessary long-range ordering, the development of a superstructure during growth of the Ta_2_O_5_ films is unlikely, as they are prepared by moderate-temperature physical vapor deposition (PVD). Nevertheless, the superstructure develops on annealing of the films in vacuum or ambient air, as discussed in the following paragraphs.

**Fig. 5:**
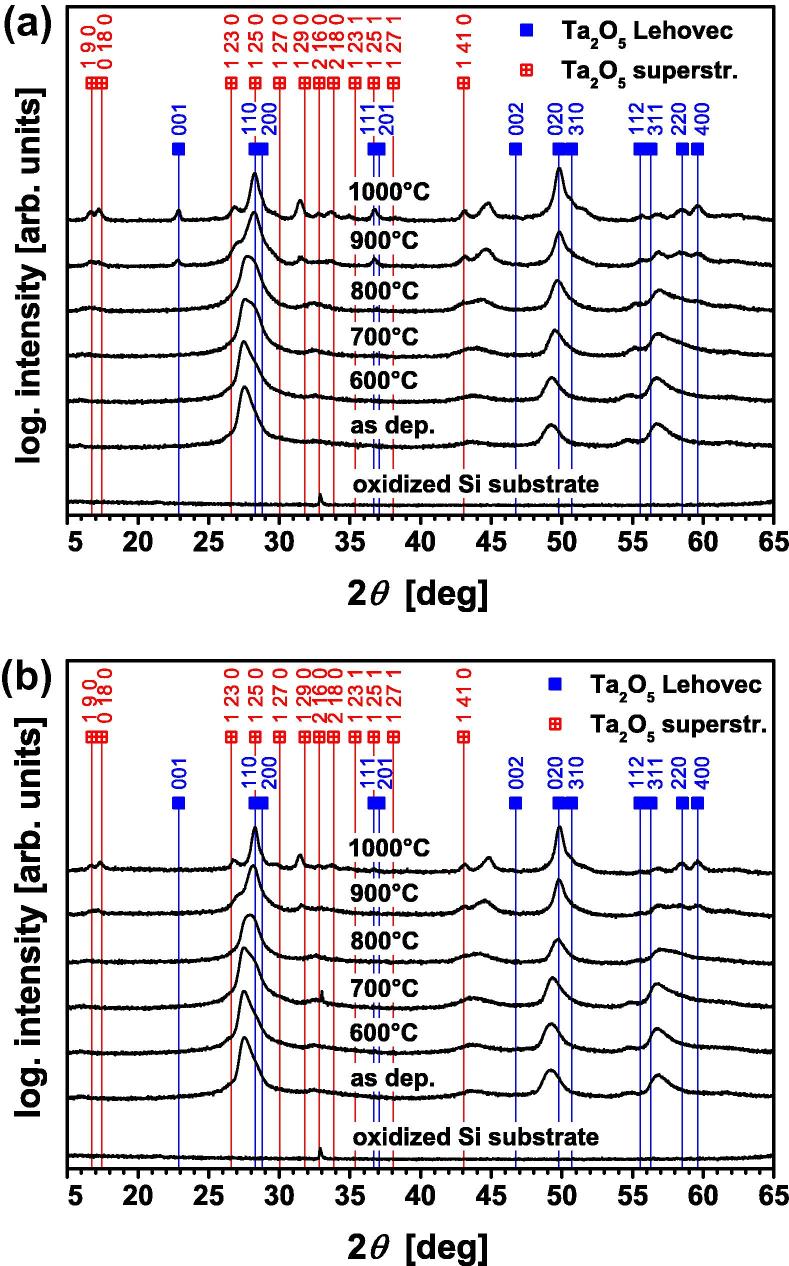
Conventional XRD pattern of the ∼15 μm thin Ta_2_O_5_ film after annealing for 1 h at temperatures up to 1000 °C in (a) vacuum and (b) ambient air.

With increasing temperature during annealing in vacuum or air (Fig. 5(a) and (b), respectively), the XRD peaks of the 15 μm thin Ta_2_O_5_ coating shift towards the indexed peak positions of the Lehovec structure (which are part of the superstructure). Additional XRD peaks can be detected for annealing temperatures >800 °C, suggesting the development of a superstructure. The XRD patterns of the films after annealing at 1000 °C exactly match the 25L superstructure published by Audier et al. [10] as well as the basic structure reported by Lehovec [7], which is a part of this superstructure. It is speculated that the additional (not indexed) peaks at diffraction angles 2*θ *⩾ 45° correspond also to the superstructure, but the corresponding peak positions at these high diffraction angles have not been published by Audier et al. [10].

**Fig. 6:**
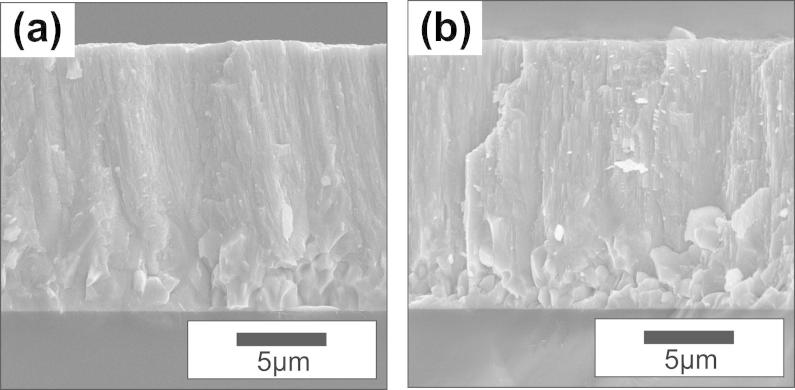
Cross-sectional SEM micrographs of the coatings deposited for 120 min after annealing for 1 h at 1000 °C in (a) vacuum and (b) ambient air.

The development of a fully crystalline structure or the increased degree of crystallinity on annealing, as suggested by XRD, can also be observed in cross-sectional micrographs of the coatings annealed at 1000 °C in vacuum (Fig. 6(a)) and air (Fig. 6(b)). In particular, the featureless region near the substrate of the as-deposited coatings (see Fig. 1) transformed into a pronounced crystalline fracture pattern as a result of the annealing treatment. This is in excellent agreement with previous results reported by Wu et al. [32].

To investigate the effect of the amorphous region and the crystalline region on the chemical composition of the Ta_2_O_5_ films, ERDA measurements of the ∼0.5 and ∼15 μm thin films were conducted. Both as-deposited samples are clearly substoichiometric, with O/Ta ratios of 2.39 and 2.33, respectively. On annealing in vacuum for 1 h at 1000 °C, the O/Ta ratio decreases even further from 2.33 to 2.23. In contrast, the corresponding annealing in air leads to an increase in the O/Ta ratio to 2.57. Irrespective of their different chemical composition on annealing in vacuum or air, no difference in their crystalline structure can be detected by XRD (compare Fig. 5(a) and (b)). This indicates that the Ta_2_O_5_ superstructures are tolerant for significant variations in the oxygen content. The ∼0.5 μm thin, mainly amorphous, coating is characterized by a hardness (*H*) of 8.2 ± 0.4 GPa and an indentation modulus (*E*) of 153 ± 17 GPa. These values increase to *H *= 14.3 ± 0.4 GPa and *E *= 188 ± 4 GPa with an increase in coating thickness to 15 μm, as thereby the crystalline fraction also increases. Their hardness only slightly decreases to 9.3 ± 0.5 and 9.4 ± 0.6 GPa, and their indentation moduli decrease to 139 ± 6 and 139 ± 3 GPa on annealing at 1000 °C in vacuum or air, respectively. The reduction in hardness and indentation modulus on annealing can be attributed to recovery and recrystallization effects, resulting in a decreased defect density and stress state [33]. The nearly identical mechanical properties of the coatings after annealing in vacuum or air, which results in a different O/Ta ratio of 2.23 and 2.57, respectively, additionally support that the Ta_2_O_5_ superstructures are tolerant for oxygen variations.

### 3.2 Calculations

**Fig. 7:**
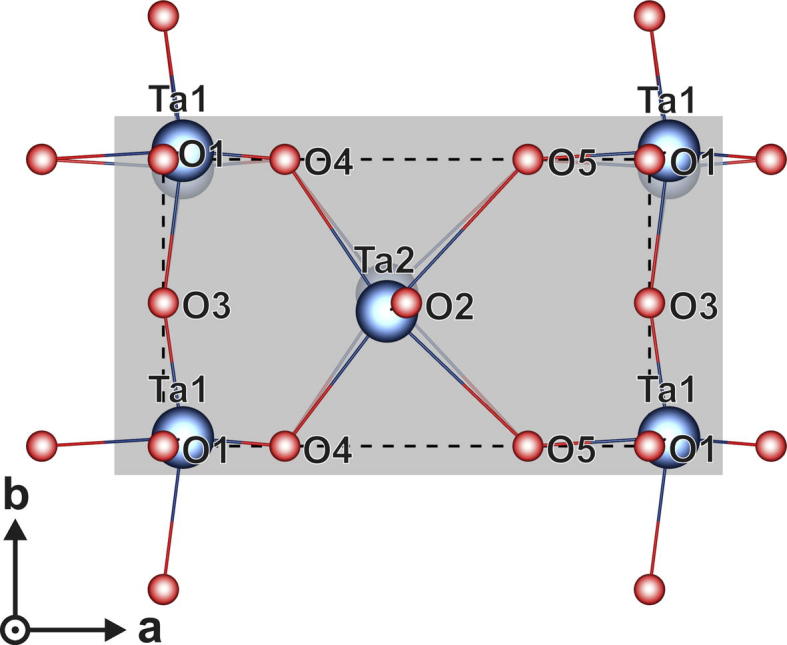
Schematic of the *c* plane for the Ta_2_O_5_ structure proposed by Lehovec [7].

Since the as-deposited films do not show any superstructure peaks, several published orthorhombic structures of Ta_2_O_5_ were selected for detailed ab initio studies, including the subtraction-type model based on U_3_O_8_ [34] and the basic-structure model described by Lehovec [7]. These are compared with the superstructure model proposed by Stephenson and Roth [11]. The orthorhombic subtraction-type model based on U_3_O_8_ is a possible basic structure with one formula unit, the structure of Lehovec has two, and the Stephenson model is a superstructure containing 11 formula units of Ta_2_O_5_ (77 atoms in the unit cell). However, the calculations show that the subtraction-type model based on U_3_O_8_ is not stable in the orthorhombic crystal structure, as it favors a monoclinic structure if a cell shape relaxation is allowed (see Table 1). A full optimization (towards lower total energy) of the crystal structure after Lehovec [7] would result in a change from the orthorhombic structure as well. Hence, the cell shape needed to be fixed during relaxation. However, by periodically alternating the occupied oxygen sites in succeeding 002-planes, the orthorhombic cell could be stabilized also during the full structural relaxation. Within the original Lehovec structure (Fig. 7), the oxygen ions denoted by O1, O2 and O3 correspond to the same type, as they are always bonded to two tantalum ions (Ta1–Ta1 or Ta2–Ta2 in the *c* direction; Ta1–Ta1 in the *b* direction). The oxygen ions O4 and O5 correspond to another type, as they are bonded to three tantalum ions (Ta1–Ta2–Ta2).

**Fig. 8:**
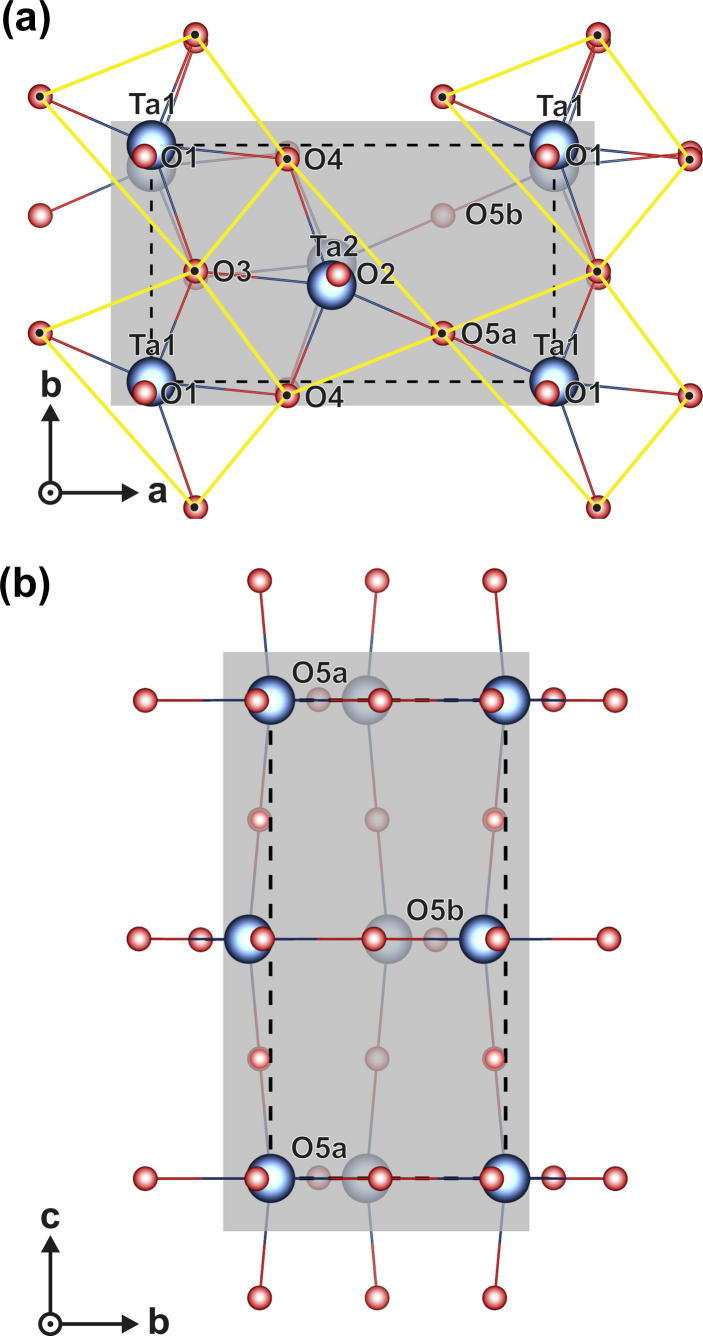
Schematic of (a) the *c* plane and (b) the *a* plane for the ab initio calculated β′-Ta_2_O_5_ structure.

In contrast, the newly developed orthorhombic description (Fig. 8) shows a different oxygen distribution. The oxygen ions O1, O2 and O5a/O5b are bonded to two tantalum ions in the *c* direction (Ta1–Ta1 or Ta2–Ta2) and also two tantalum ions in the *a*–*b* direction (Ta1–Ta2). The oxygen ions O3 and O4 are three-coordinated to tantalum (Ta1–Ta1–Ta2 or Ta2–Ta2–Ta1) (Fig 8(a)). Although the fully relaxed structure is similar to that of Lehovec, there is an important difference in the oxygen ion position labeled O5. For the model, the occupied oxygen position periodically alternates between O5a and O5b in succeeding 002-planes. For simplicity, the new description is called β′-Ta_2_O_5_, which addresses the relation with the Lehovec model and the condition that one oxygen ion periodically occupies either position O5a or position O5b. The lattice parameters and the ion positions of the fully relaxed β′-Ta_2_O_5_ structure are given in Table 2. The new β′-Ta_2_O_5_ structure can also easily be described by the space group 53 (*Pncm*) with the Wyckoff positions as presented in Table 3. The lattice positions thereby obtained exhibit a maximum deviation of only 0.0056 Å from the ion positions presented in Table 2. Consequently, they can be seen as identical.

**Table 2:** Ion positions of the orthorhombic β′-Ta_2_O_5_ structure with lattice parameters for two formula units in the *c* direction of *a* = 6.425 Å, *b *= 3.769 Å and *c *= 7.706 Å.

Ion	*x*	*y*	*z*	Occupancy
Ta1	0.00	0.00	0.00	1
Ta2	0.45	0.41	0.00	1
Ta3	0.00	0.91	0.50	1
Ta4	0.45	0.50	0.50	1
O1	0.98	0.95	0.25	1
O2	0.46	0.45	0.25	1
O3	0.11	0.47	0.00	1
O4	0.34	0.94	0.00	1
O5a	0.72	0.20	0.00	1
O6	0.98	0.95	0.75	1
O7	0.47	0.45	0.75	1
O8	0.11	0.44	0.50	1
O9	0.34	0.97	0.50	1
O5b	0.72	0.70	0.50	1

**Table 3:** Wyckoff positions for β′-Ta_2_O_5_ within space group 53 (*Pncm*) with lattice parameters of *a* = 6.425 Å, *b *= 3.769 Å and *c *= 7.706 Å.

Ion	*x*	*y*	*z*	Occupancy
Ta	0.7760	0.7955	0.0000	1
O	0.5000	0.0000	0.0000	1
O	0.8860	0.2630	0.0000	1
O	0.2425	0.2500	0.2500	1

The small but significant difference between the model and that of Lehovec is further illustrated when calculating the local O/Ta ratio *f*_ox_ for specific Ta ions:

(1) $$f_{\text{ox}}=\overset{n}{\underset{i=1}{\sum }}\frac{1}{x_{i}}$$

where *n* is the number of O ions surrounding the considered Ta ion, and *x* is the coordination of O ions to Ta ions. Local O/Ta ratios of *f*_ox_ = 2.66 around Ta1 and *f*_ox_ = 2.33 around Ta2 ions clearly reveal two different local neighborhoods for the Lehovec model. Contrarily, the fully relaxed β′-Ta_2_O_5_ model yields exactly *f*_ox_ = 2.5 for Ta1 as well as for Ta2. Consequently, all Ta positions are chemically equivalent and stoichiometric. This can only be achieved by the periodic alternation of the occupied oxygen site between position O5a and O5b in succeeding 002-planes (see also Fig. 8(b)). This configuration can conveniently be described by double-pyramidal building blocks with irregular quadrilateral base planes, as visualized in Fig. 8(a) by yellow lines. These building blocks form zigzag chains in the *b* direction, and they are connected to neighboring chains via O5a ions and O5b ions in succeeding 002-planes and vice versa.

Since the as-deposited crystalline coatings are not fully stoichiometric, sub-stoichiometric configurations of the β′-Ta_2_O_5_ model with an O/Ta ratio of 2.33 were also considered. The formation of oxygen vacancies is energetically more favorable than the generation of interstitial tantalum ions or the substitution of oxygen by tantalum ions. Also, the cell size needed for obtaining an O/Ta ratio of 2.33 is much smaller for the case of oxygen vacancies. In the case of additional interstitial tantalum ions, 7 unit cells of β′-Ta_2_O_5_ would be necessary, whereas only 3 unit cells are necessary for the case of oxygen vacancies. Therefore, the ab initio studies were focused on oxygen vacancies, for which a supercell with three times the β′-Ta_2_O_5_ model in the *b* direction was constructed, hence containing six times the formula unit. Oxygen vacancies O1 and O2 were not considered, as they correspond to the most stable positions. The ab initio calculations show that the orthorhombic structure is only stable if the oxygen vacancies are placed at 3-coordinated oxygen positions, which are O3 and O4. Thereby, the lattice parameters change from *a *= 6.425 Å, *b *= 3.769 Å and *c *= 7.706 Å to *a *= 6.462 Å, *b *= 3.722 Å and *c *= 7.654 Å, respectively (see Table 1). Both structures, the stoichiometric as well as the substoichiometric β′-Ta_2_O_5_ structure, better fit the experimental structure analysis by XRD than the Lehovec model, which is used to index the individual peak positions in Fig. 2, Fig. 5. This is also valid for the X-ray nano-diffraction analysis across the coating thickness, where, in particular, the 111 and 201 diffraction peaks at azimuth angles of *δ* = 0° and 45° (Fig. 4) are better described by the β′-Ta_2_O_5_ structure. Based on these results, it is suggested that a significant portion of the sputtered Ta_2_O_5_ thin films crystallize in the β′-Ta_2_O_5_ structure.

**Fig. 9:**
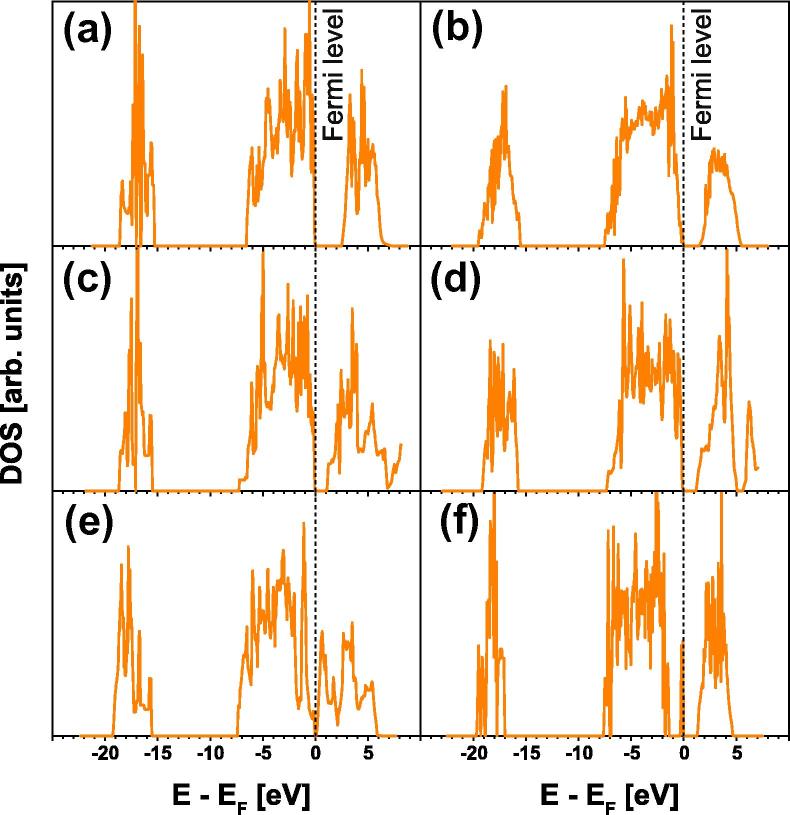
Ab initio calculated DOS for (a) the β′-Ta_2_O_5_ structure, (b) the orthorhombic superstructure after Stephenson and Roth [11], (c) the hexagonal structure after Fukumoto and Miwa [31], (d) a monoclinic structure (relaxed orthorhombic subtraction-type-model based on U_3_O_8_[34]), (e) the orthorhombic structure after Lehovec [7] and (f) the oxygen-deficient β′-Ta_2_O_5_ for obtaining an O/Ta ratio of 2.33.

The density of states (DOS) of the various Ta_2_O_5_ structures provides further evidence for the β′-Ta_2_O_5_ model. In particular, with the band-gap of ∼2.5 eV, Fig. 9(a) is much closer to experiments (∼4 eV [2], [35], [36]) than the values obtained for the other structures. The Stephenson superstructure yields a band-gap of ∼1.5 eV (Fig. 9(b)), the hexagonal low-temperature phase (δ-Ta_2_O_5_) and the monoclinic structure (obtained by a complete relaxation of the subtraction-type-model based on U_3_O_8_ [34]) yield even smaller band-gaps (see Fig. 9(c) and (d), respectively). The orthorhombic structure by Lehovec hardly exhibits any band gap (Fig. 9(e)) and is actually only stable during relaxation if the cell shape is fixed. As the underestimation of band-gaps by GGA is a well-known deficiency of standard DFT, the results for the β′-Ta_2_O_5_ model suggest excellent agreement with experiments. Introducing oxygen vacancies to the β′-Ta_2_O_5_ model (according to the above described procedure, resulting in an O/Ta ratio of 2.33) causes no significant change in the calculated band-gap (see Fig. 9(f)), but leads to defect-induced electronic states in the middle of the band-gap. This was recently also observed for the Stephenson superstructure [37]. A similar model to the β′-Ta_2_O_5_ structure was recently published by Wu et al. [36]. However, their model yields *f*_ox_ values different from 2.5 for the individual tantalum ions, which probably explain their much smaller GGA band-gap <0.5 eV.

The energies of formation (*E*_f_) of the discussed structures (Table 1) exhibit the largest negative value for the Stephenson superstructure with −3.259 eV atom^−1^. However, their formation requires kinetic activity and long-range ordering processes, which are often not accessible during fabrication, especially when using low- or medium-temperature PVD techniques such as for the Ta_2_O_5_ coatings (sputter deposited at 500 °C). Among the basic-structure types investigated and reported, the proposed β′-Ta_2_O_5_ structure (composed of 14 atoms) provides the largest negative *E*_f_ value with −3.209 eV atom^−1^. This only slightly decreases to −3.146 eV atom^−1^, if oxygen vacancies are introduced to allow the formation of substoichiometric Ta_2_O_5_ composites (with O/Ta = 2.33). The hexagonal low-temperature phase (δ-Ta_2_O_5_), the monoclinic structure, as well as the Lehovec structure show considerably less negative energies of formations with *E*_f_ = −3.020, −2.962 and −2.947 eV atom^−1^, respectively, and hence they are significantly less favorable.

## 4 Summary and conclusions

Tantalum pentoxide Ta_2_O_5_ thin films were prepared by reactive sputter deposition at 500 °C for detailed studies of their growth morphology, structure and mechanical properties. X-ray nano-diffraction studies across the ∼15 μm coating thickness exhibited a nearly amorphous region for the first 0.5 μm to be followed by a pronounced 110–200 growth orientation. Owing to limited kinetic activity and limited long-range order processes during the deposition of the films, only an orthorhombic basic structure developed, while no trace of a superstructure was found. The latter, which is the stable configuration for crystalline Ta_2_O_5_, only develops in the films if they are annealed at temperatures >800 °C in vacuum or air. Although the annealing treatment in vacuum or air leads to a change in the chemical composition from the as-deposited O/Ta ratio of 2.33 to 2.23 and 2.57, respectively, the structure is almost identical. In combination with the almost identical hardnesses of 9.3 ± 0.5 and 9.4 ± 0.6 GPa and indentation moduli of 139 ± 6 and 139 ± 3 GPa after these treatments, this suggests that the Ta_2_O_5_ structure is flexible for slight oxygen variations.

Based on ab initio calculations, one can conclude that the as-deposited structure is best described by a modified orthorhombic Lehovec structure. Starting from the Lehovec structure, the new model is based on two formula unit blocks in the *c* direction, hence 14 atoms, but varies in the occupied oxygen sites in the 002-planes. In the model, the occupied oxygen site periodically alternates between two possible positions in succeeding 002 planes. Thereby, the local O/Ta ratio of any Ta position is always 2.5. This is a unique configuration among all structures investigated. Further evidence for the β′-Ta_2_O_5_ is provided by the energy of formation and density of states. Among all non-superstructure models, the β′-Ta_2_O_5_ structure exhibits the most negative value for the energy of formation with −3.209 eV atom^−1^ and a band-gap of 2.5 eV, which is closest to the experimental value of 4 eV. Actually, β′-Ta_2_O_5_ is almost as stable as the well-accepted orthorhombic Stephenson superstructure yielding *E*_f_ = −3.259 eV atom^−1^, for which long-range ordering of 77 atoms in the unit cell is necessary.

Consequently, the description of a metastable orthorhombic Ta_2_O_5_ structure, with lattice parameters of *a *= 6.425 Å, *b *= 3.769 Å and *c *= 7.706 Å, is ideal for materials without long-range ordering (and the thereby missing formation of a superstructure) as well as for detailed atomistic studies, owing to the much smaller necessary cell sizes. The β′-Ta_2_O_5_ structure proposed here can easily be described by space group 53 (*Pncm*) with four Wyckoff positions.
